# Positron Emission Tomography-Derived Radiomics and Artificial Intelligence in Multiple Myeloma: State-of-the-Art

**DOI:** 10.3390/jcm12247669

**Published:** 2023-12-13

**Authors:** Luigi Manco, Domenico Albano, Luca Urso, Mattia Arnaboldi, Massimo Castellani, Luigia Florimonte, Gabriele Guidi, Alessandro Turra, Angelo Castello, Stefano Panareo

**Affiliations:** 1 Medical Physics Unit, Azienda USL of Ferrara, 45100 Ferrara, Italy; luigi.manco@ausl.fe.it (L.M.); a.turra@ospfe.it (A.T.); 2 Nuclear Medicine Department, University of Brescia and ASST Spedali Civili di Brescia, 25123 Brescia, Italy; domenico.albano@unibs.it; 3 Department of Translational Medicine, University of Ferrara, 44121 Ferrara, Italy; luca.urso@unife.it; 4 Nuclear Medicine Unit, Fondazione IRCCS Ca’ Granda, Ospedale Maggiore Policlinico, 20122 Milan, Italy; mattia.arnaboldi@policlinico.mi.it (M.A.); massimo.castellani@policlinico.mi.it (M.C.); luigia.florimonte@policlinico.mi.it (L.F.); 5 Medical Physics Unit, University Hospital of Modena, 41125 Modena, Italy; guidi.gabriele@aou.mo.it; 6 Nuclear Medicine Unit, Department of Oncology and Hematology, University Hospital of Modena, Via del Pozzo 71, 41124 Modena, Italy; panareo.stefano@aou.mo.it

**Keywords:** radiomics, artificial intelligence, AI, machine learning, deep learning, multiple myeloma, positron emission tomography, PET

## Abstract

Multiple myeloma (MM) is a heterogeneous neoplasm accounting for the second most prevalent hematologic disorder. The identification of noninvasive, valuable biomarkers is of utmost importance for the best patient treatment selection, especially in heterogeneous diseases like MM. Despite molecular imaging with positron emission tomography (PET) has achieved a primary role in the characterization of MM, it is not free from shortcomings. In recent years, radiomics and artificial intelligence (AI), which includes machine learning (ML) and deep learning (DL) algorithms, have played an important role in mining additional information from medical images beyond human eyes’ resolving power. Our review provides a summary of the current status of radiomics and AI in different clinical contexts of MM. A systematic search of PubMed, Web of Science, and Scopus was conducted, including all the articles published in English that explored radiomics and AI analyses of PET/CT images in MM. The initial results have highlighted the potential role of such new features in order to improve the clinical stratification of MM patients, as well as to increase their clinical benefits. However, more studies are warranted before these approaches can be implemented in clinical routines.

## 1. Introduction

Multiple myeloma (MM) is a hematologic malignancy, accounting for approximately 10% of all blood tumors. It is associated with the monoclonal proliferation of plasma cells, resulting in the abnormal production of immunoglobulins in the bone marrow (BM) [1,2]. MM diagnosis is based on the so-called “CRAB criteria”, which include hypercalcemia (C), renal damage (R), anemia (A), and lytic bone lesions (B), along with at least 10% of pathological infiltration on BM examination [3]. The NCCN guidelines recommend either whole-body (WB) low-dose computed tomography (CT) or [^18^F]1fluorodeoxyglucose (FDG) positron emission tomography (PET)/CT for the initial diagnosis, staging, evaluation of treatment outcomes, and follow-up [4]. WB-magnetic resonance imaging (MRI) might be helpful in the case of diffuse BM infiltration and to evaluate the axial skeleton, particularly for localizing spinal cord and/or nerve root compression for surgery or radiotherapy [5].

In this context, the identification of noninvasive, reliable, reproducible imaging tools is of utmost importance to allow a patient-tailored medical approach, particularly in cases of heterogeneous diseases like MM. The Italian Myeloma Criteria for PET Use (IMPeTUs), based on the Deauville criteria, have been proposed to standardize the interpretation of PET/CT in patients with MM [6]. Nevertheless, the visual assessment of BM in some cases is difficult; therefore, these criteria still remain limited in clinical practice. Moreover, the introduction of new therapeutic agents, such as chimeric antigen receptor T-cell therapy (CAR-T therapy), teclistamab, or talquetamab-tgvs (bispecific antibodies that bind both an antigen on T cells of the patients and an antigen on the myeloma cells) has generated new pattern of response often not sufficiently interpretable by the traditional imaging criteria [7].

A possible answer to this unmet clinical need might be found using radiomics analysis. This technique is rapidly wide-spreading in the evidence from recent literature, as it allows additional information to be mined from medical images beyond the human eyes’ resolving power. Indeed, radiomics analysis can be performed on medical images from different modalities, allowing for an integrated cross-modality approach using the potential additive value of extracted imaging information, such as from MRI, CT, and PET, instead of evaluating each modality on its own [8]. These additional data could be used to obtain a greater knowledge of the molecular and genotypic characteristics of tumor lesions in order to guide treatment selection and improve outcomes [9,10,11]. Machine learning (ML) is a branch of artificial intelligence in which, based on the training datasets that are first provided, the computer develops its own logic for answering future questions. In addition, deep learning (DL) employs multiple layers of neural networks, leading to expanded “neuronal” complexity to significantly enhance the computational power. ML and DL algorithms use different learning approaches [12,13]. These procedures are aimed at predicting response variables in one or more classes in clinical questions related to different situations, such as diagnosis, segmentation, and clinical outcomes. The basic rationale is that starting from a dataset, specific training of the classifier produces a classification performance of a case that is unknown to the starting dataset. Supervised learning techniques are, nowadays, the most widely used methods in ML and those with the best results. In this data science strategy, the algorithm uses training inputs and their corresponding outputs in order to learn a rule that connects inputs to outputs. Another common approach provides unsupervised learning, and no referenced outputs are used by the algorithm to find a structure in the inputs. Finally, the algorithm classes are completed using reinforcement and transfer learning [14]. 

The aim of our systematic review is to summarize the current evidence of PET radiomics and AI models in MM, describing their drawbacks as well as their potential applications in clinical practice to improve patients’ management.

## 2. Materials and Methods

### 2.1. Literature Search Strategy

A search on three authoritative databases and online sources (Pubmed/Medline, Web of Science, Scopus) was performed running the multiple queries: “(myeloma AND (radiomics OR radiomic) AND pet) NOT review”,”(myeloma AND (machine AND learning) AND pet) NOT review”,”(myeloma AND (deep AND learning) AND pet) NOT review”,”(myeloma AND (artificial AND intelligence) AND pet) NOT review”. English-language original articles published before 1 July 2023 were considered. The references of the selected studies were screened to identify any additional relevant literature to include.

### 2.2. Study Selection, Data Collection, and Quality Assessment

Titles and abstracts were independently reviewed by three authors (L.U.; L.M.; and A.C.) to select the studies to include. Full articles were retrieved when the abstract was considered relevant. Inclusion criteria applied during selection were as follows: (a) articles concerning smoldering myeloma or MM; and (b) articles on texture analysis and AI applications derived from PET/CT, PET/MRI. The following papers were considered ineligible: (a) review articles, conference papers, case reports, editorials, case series, letters to the editor; (b) articles not in the English language; (c) studies not within the field of interest (i.e., not radiomics/AI aims, not PET images, and not MM); (d) preclinical studies. The data were summarized in a database using the following fields: first author, journal, year, title, exclusion/issues, imaging modality, computer science area, number of patients, training set size, test set size, validation set size, and the setting/purpose of the study (diagnosis, prognosis, pattern distribution, and differential diagnosis) for the subsequent data analysis.

For each study, the radiomic analysis was assessed based on the radiomics quality score (RQS 2.0) introduced by Lambin and colleagues in 2017 [15] to specifically evaluate the quality of reporting in the radiomics context. For a robust calculation, RQS 2.0 was blindly computed by two of the authors, one nuclear medicine physician (L.U.) and one medical physicist (L.M.), respectively; both have at least 3 years of experience in radiomics. Discrepancies were discussed to reach a consensus. 

QUADAS-2 was used as tool for assessing risk of bias in individual studies and concerns regarding applicability [16]. Quality assessment was independently carried out by two authors, and any discrepancy was solved by a third author.

## 3. Results

**Figure 1:**
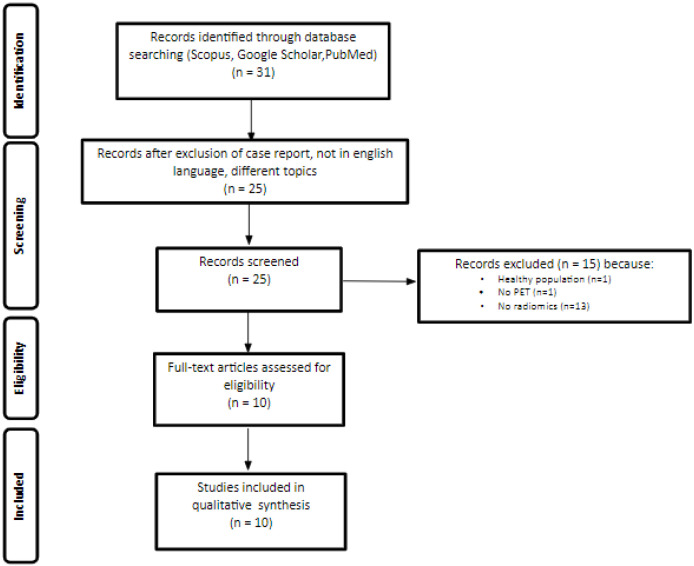
PRISMA flowchart of study selection and inclusion in the systematic review.

The PRISMA flowchart of the articles included in our review is reported in Figure 1. The literature search identified a total of 31 studies. According to the aforementioned eligibility criteria, 10 studies were included [17,18,19,20,21,22,23,24,25,26]. Among these, eight studies were retrospective and two prospective. Furthermore, only one study was performed using [^68^Ga]Pentixafor [26], while all the others used [^18^F]FDG as the radiotracer (Table 1). 

### 3.1. Radiomics Assessment

The RQS 2.0 is reported in Table 2. Of the 10 included papers, 9 qualified as conventional radiomics approaches, and in 1, a DL approach was performed [26]. Within the conventional (handcrafted) radiomics approach, semiautomated segmentation was the most common strategy for VOI identification (seven studies), followed by two involving totally manual delineation [22,24].

All selected radiomic studies performed a statistical analysis in order to select the optimal/robust features. The total number of features initially extracted from each VOI, both for the CT and PET datasets, is summarized in Table 2 and ranged from 15 [25] to 1702 [19]; the most common features were SUVmax, shape, and first-order statistics, followed by textural features. After analysis, the selected features ranged from 3 [19] to 457 [22]. The least absolute shrinkage and selection operator (LASSO) and Mann–Whitney U test were the most popular approaches for this task (three studies for each one). R-Sofware [17,19,22] and Python [22,25,26] were the most used AI open source programming languages for the endpoint prediction and statistical analysis. In addition, Orange [18,21] was the most used toolkit to build the predictive model. The main data-mining algorithms used to perform ML strategies were random forests, including their extension in random survival forest [17,18,20,21,23,25] and k-nearest neighbors [18,20,21,23,26]. The first two mentioned classifiers belong to the tree-based class. Tree-based ML methods are among the most commonly used supervised learning methods. In these, algorithm branches and nodes are constructed by recursively splitting a training sample using different features from a dataset. The splitting is based on learning simple decision rules inferred from the training data. k-nearest neighbors is an instance-based algorithm using a kernel function to build the hypothesis from the training data. Only one paper [24] did not develop a model prediction based on radiomics signatures. The study of Xu et al. [26], the only one classified as deep learning, adopted three different models, using two convolutional neural networks (CNNs), V-Net and W-Net, to segment and detect lesions. Data augmentation was used to generate patches. Yet, they outperformed traditional ML methods, such as the random forest classifier (RF), k-nearest neighbors (k-NN), and the support vector machine (SVM). In addition, internal validation was performed in 8 out of 10 studies using a fold-cross validation procedure [17,18,20,21,22,23,25,26]. Table 3 summarizes studies’ data mining.

Finally, regarding the RQS2.0 evaluation, all the considered studies ranged between 22.73% and 59.09%.

### 3.2. Quality (Risk of Bias) Assessment

**Figure 2:**
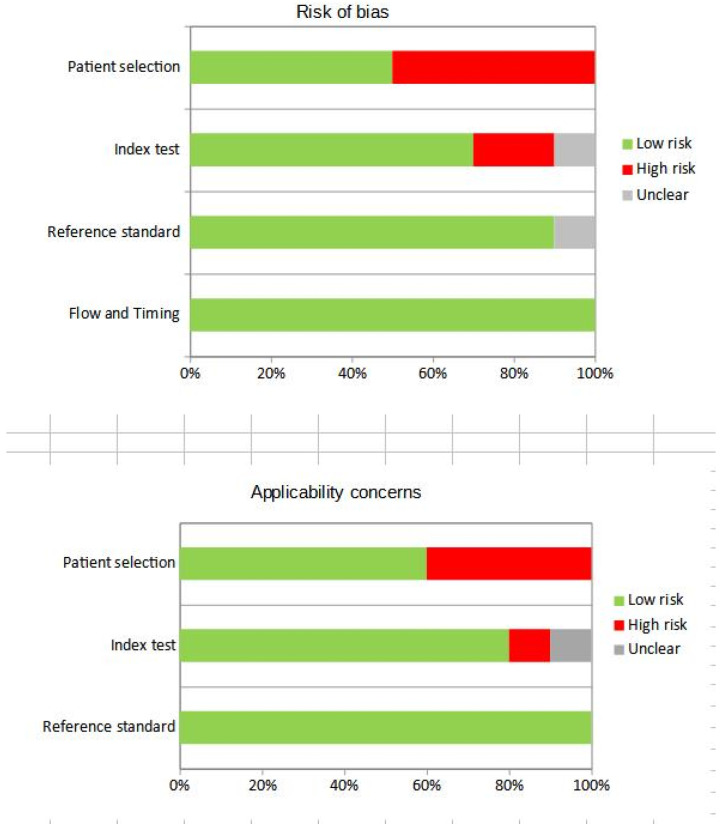
Overall quality assessment using QUADAS-2 tool. Studies included in the systematic review are classified as at low risk or high risk of bias or applicability concerns for different domains.

The overall evaluation of the risk of bias and concerns regarding the applicability of the studies included in the systematic review according to QUADAS-2 is presented in Figure 2.

### 3.3. Diagnosis

The initial diagnosis of MM diffuse disease can be challenging, although [^18^F]FDG PET/CT is potentially able to improve the MM risk stratification by detecting medullary and extramedullary focal lesions. In their prospective study, Mesguich et al. [23] evaluated the role of PET/CT radiomics in the diagnosis of MM diffuse disease. In a cohort of 30 patients, 20 were randomly assigned to a training set and 10 to an independent test set, while MRI was considered the reference standard. Overall, 5 out of 174 radiomics features (87 from both CT and PET) were selected using random forest and correlation analysis. The visual analysis of the PET/CT images determined a sensitivity, specificity, and accuracy of 67%, 75%, and 70%, respectively. On the other hand, the radiomics analysis reached a sensitivity, specificity, and accuracy of 93%, 86%, and 91% on the training set, whereas on the independent test, sensitivity, specificity, and accuracy were 100%, 70%, and 80%, respectively. These preliminary results supported the potential role of radiomics to identify diffuse bone marrow infiltration in myeloma patients.

Xu et al. [26] developed two CNN-based deep learning methods, i.e., V-Net and W-Net, for the identification of bone lesions using [^68^Ga]Pentixafor PET/CT. After the first evaluation on digital phantoms, the preliminary results were also encouraging on the real PET/CT of MM patients, achieving a specificity as high as 99.68%. Furthermore, V-Net outperformed traditional machine learning methods, such as RF, k-NN, and SVM. 

### 3.4. Differential Diagnosis between Myeloma and Bone Metastases

Although MM and bone metastases share the same site of occurrence, clinical manifestations, and imaging characteristics, they have a different pathogenesis, and their correct identification affects quality of life and patient prognosis. Recently, Jin et al. [22] aimed to examine whether [^18^F]FDG PET/CT radiomics analysis could improve the diagnostic accuracy between MM and BMTS in 131 patients. Overall, 279 radiomics features were extracted, and three classification models, such as CT, a PET−based model, and ComModel (PET model + SUVmax), were constructed and compared. All three models achieved a high performance both in the training (AUC = 0.909, 0.949, and 0.973, respectively) and validation sets (AUC = 0.897, 0.929, and 0.948, respectively). Moreover, both the ComModel and the PET model showed significant differences in the classification diagnosis of MM and BMTS compared to human experts and SUVmax. The same aim was pursued in the study of Mannam and colleagues [20], who extracted 138 PET and 138 CT radiomics features in a cohort of 20 patients with MM and 20 with BMTS. However, the machine learning models using CT parameters better-differentiated MM from BMTS than models using PET features, although the combined models using PET and CT data were superior to those using single data.

### 3.5. Therapy Response Assessment and Prognosis

Radiomics data from [^18^F]FDG PET/CT in patients affected by MM have also been evaluated as prognostic factors of survival [17,19,24,25]. Zhong et al. [17] investigated the different combination image data of PET, CT, clinical parameters, and six machine learning algorithms for predicting prognosis in MM patients. Overall, five PET−derived features, four CT-derived features, and six clinical features were significantly associated with PFS and included in the model construction. Moreover, the performance of PET and CT models combined with clinical variables was significantly improved in various algorithms, paving the way for better patient selection for precision therapy. 

Likewise, Ni et al. [19] developed three different models to predict prognosis in 98 newly diagnosed MM patients. Also, in this study, by combining PET/CT-derived radiomics features and clinical data, a higher prognostic performance than models with radiomics features or clinical data alone was achieved. 

In a retrospective study of 45 patients with smoldering myeloma, Ripani et al. [24] showed that several texture features were significantly associated with progression to symptomatic MM and a shorter survival. Recently, in a large cohort of 139 MM patients enrolled in two prospective clinical trials, Jamet et al. [25] explored the prognostic value of PET−derived radiomics features through a machine learning algorithm, the so-called random survival forest (RSF). Three out of twenty-two radiomics features were selected using the RSF approach, and two different prognostic groups were defined with a mean hazard ratio of 4.3 ± 1.5. 

Measurable residual disease (MRD) after systemic treatment is one of the most important prognostic parameters in MM patients. Currently, MRD is assessed by combining multiparameter flow cytometry (MFC) or next-generation sequencing (NGS) from bone marrow biopsy/aspiration with a visual analysis of [^18^F]FDG PET/CT, although with controversial results [6]. In order to improve the diagnostic accuracy and quantification of MRD, Milara et al. [21] proposed and developed a software tool for the extraction of radiomic features from the [^18^F]FDG PET/CT images of 39 patients. This tool was based on the creation of a skeleton mask on CT images, where spinal canal and compact bone were removed, and then the mask was used to segment the bone marrow from the PET images. The SUV parameters, as well as radiomic features from GLCM, GLRLM, and NGTDM, were extracted and evaluated for differentiating PET+/PET− and MFC+/MFC− groups. Of note, 22 out of 29 features showed statistically significant differences and variance; energy and entropy were those with the lowest *p*-value to discriminate between PET+ and PET−. On the other hand, only one radiomic feature was significant to differentiate MFC+ and MFC−. Furthermore, seven machine learning algorithms for PET+/PET− and MFC+/MFC− classification were also assessed, demonstrating acceptable results only for PET+/PET− differentiation.

One of the limits of the abovementioned study is related to the whole bone marrow segmentation, which hinders the relationship with the MFC results derived from small and specific biopsy sites. Therefore, the same group [18] estimated and quantified, using radiomics analysis, the representativeness of a single bone marrow biopsy from [^18^F]FDG PET/CT. Afterward, they compared such features with those extracted from the whole bone marrow and to the MFC results. Similar to the previous study, 16 features were statistically significant to differentiate PET+/PET−, such as SUVmax, the gray-level nonuniformity or entropy; in addition, the machine learning algorithms demonstrated performances as high as 0.974 in PET+/PET− classification but not for MFC+/MFC− differentiation. In conclusion, the results demonstrated the validity and robustness of the sample sites and radiomics features from [^18^F]FDG PET images for the evaluation of MRD.

## 4. Discussion

MM is the second most prevalent hematologic neoplasm, developing from the monoclonal gammopathy of undetermined significance to active MM through an intermediate state called smoldering MM. At present, [^18^F]FDG PET/CT has achieved a primary role for diagnosis and prognosis determination, as well as for treatment response assessment and, in general, for clinical decision making [27,28]. 

Moreover, in an era where personalized patient care is constantly enhancing, the introduction of radiomics and AI can have a potential high impact on noninvasive patients’ stratification due to their ability to extract pertinent clinical information from a large volume of data generated within the clinical routine [9,10,29]. As a matter of fact, in the United States, the number of AI/ML-enabled medical devices approved by the Food and Drug Administration has progressively increased, accounting for 691, approximately 15% in the only 2023. Furthermore, radiology and nuclear medicine are emerging as the predominant fields for the application of AI/ML-enabled medical devices, allowing the accumulation of useful data for medical research [30].

While several studies have been published regarding the role of AI for solid malignancies, only a few papers have assessed the accuracy of the radiomics features and ML algorithms from [^18^F]FDG PET/CT images in MM patients in different clinical scenarios [17,18,19,20,21,22,23,24,25,26,31]. Promising results have been obtained for the characterization of myeloma lesions [20,22,23,26]. At present, the initial diagnosis of MM diffuse infiltration disease from PET/CT images is based on a visual approach using the spleen or liver uptake as the cut-off, which is not free from limitations. If validated in larger studies, radiomics could provide an additional tool to identify diffuse disease using PET/CT, allowing the better-selection of MM high-risk patients and improving subsequent therapeutic evaluation [23]. 

Correct discrimination between MM lesions and bone metastases is of crucial importance as it implies different treatment options. In this context, radiomics models have showed high diagnostic efficacy in classifying these two diseases, with an AUC value up to 0.973 [20,22]. Although preliminary, these results pave the way for a new possibility of noninvasive assessment of the lesion and its heterogeneity.

Although most studies were conducted using [^18^F]FDG, deep learning methods from whole-body [^68^Ga]Pentixafor PET/CT images have also demonstrated valuable results for MM lesion detection [26].

To date, the most interesting application of PET radiomics and AI in MM appears to be patients’ prognostic stratification. In particular, it emerges that the combination of either radiomics or ML models with clinical data significantly improved prognostic performance rather than the traditional single models [17,19,25]. Furthermore, the texture features derived from PET images were significantly associated with progression from smoldering to symptomatic MM, as well as with shorter survival, leading to the early identification of patients at a higher risk of evolving disease [26]. 

Finally, MRD represents one of the most important biomarkers to identify patients with longer responses and better outcomes. At present, the results from a combination of bone marrow biopsy with PET/CT visual analysis are commonly inconsistent; therefore, the correct identification of MRD remains limited [32,33,34]. Milara and colleagues have demonstrated the high accuracy of radiomics features for MRD assessment, although in a small and unbalanced cohort of patients with MM [18,21].

Some limitations of our review should be underlined. First of all, as most of the studies included are of a single-center retrospective design [17,18,19,20,21,22,24,26], there could have been accidental bias in the patient selection process. Secondly, they often included a relatively small patient cohort. Regarding the radiomics characteristics, all the included studies in this review achieved a low RQS, where only one [25] scored more than 50%. This reflects the way the RQS is structured. Indeed, in addition to some technical aspects of the studies’ design, such as data harmonization and statistical optimization, the higher score is due to the trial international multicenter design and model validation on external cohorts. This is a strong point that ensures data homogeneity and the higher external reproducibility of the studies. In this context, two radiomics guidelines have been proposed in order to improve the harmonization across studies from different centers, the so-called “Image Biomarker Standardization Initiative” and the ComBat method [35,36]. The first aims to produce and validate reference values for radiomics features, while the second has been proposed to deal with the “center-effect”, in other words to reduce the variations of scanner models, reconstruction algorithms, and acquisition protocols, which are frequently unavoidable in multicenter studies.

## 5. Conclusions

Our review demonstrates that PET/CT-based radiomics models associated with ML and algorithms can be a valuable tool to improve the clinical stratification of MM patients and to increase their clinical benefits. Nevertheless, despite the big effort conducted over the last five years, more studies are warranted before these approaches can be implemented as part of the clinical routine.

## Figures and Tables

**Table 1:** Summary of general characteristics of studies.

Author	Year	Design	Setting/Aim	Imaging	Number of Patients
Zhong et al. [17]	2023	R	Diagnosis	FDG-PET/CT	98
Milara et al. [18]	2023	R	Therapy response assessment	FDG-PET/CT	39
Ni et al. [19]	2023	R	Prognosis	FDG-PET/CT	98
Mannam et al. [20]	2022	R	Myeloma vs. MTS	FDG-PET/CT	40
Milara et al. [21]	2022	R	Therapy response assessment	FDG-PET/CT	39
Jin et al. [22]	2022	R	Myeloma vs. MTS	FDG-PET/CT	131
Mesguich et al. [23]	2021	P	Diagnosis	FDG-PET/CT	30
Ripani et al. [24]	2021	R	Pattern distribution	FDG-PET/CT	45
Jamet et al. [25]	2020	P	Prognosis	FDG-PET/CT	139
Xu et al. [26]	2018	R	Diagnosis	[^68^Ga]Pentixafor-PET/CT	12

MRD: measurable residual disease; MTS: metastasis; P: prospective; R: retrospective.

**Table 2:** Summary of the studies’ radiomic features analyses.

Author	TA	FTs	FT Types	Sw TA	Sw Class	Selected FTs	Statistical Test	RQS 2.0 (%)
Zhong et al. [17]	Yes	408 (CT) 266 (PET)	HSLM, GLCM, GLDM, GLRLM, GLSZM, NGTDM	LifeX	OS	4 (CT) 5 (PET)	Univariable Cox regression and LASSO	18 (27.27%)
Milara et al. [18]	Yes	32 (PET)	HSLM, GLCM, GLRLM, NGTDM	Matlab	C	19/28 * (PET)	MW, SC	20 (30.30%)
Ni et al. [19]	Yes	1702 (CT and PET)	SH, FO, GLCM, GLSM, GLSZM, NGTDM, GLDM	3D slicer	OS	3 (PET)	LASSO and 10-FCV proportional-hazards model	18 (27.27%)
Mannam et al. [20]	Yes	138 (CT) 138 (PET)	SH, FO, GLCM, GLRLM, GLDM, NGTDM, GLSZM, Wavelat	3D slicer	OS	5 (CT) 5 (PET)	ROC	24 (36.36%)
Milara et al. [21]	Yes	29 (PET)	SUVmax, GLCM, GLRLM, NGTDM	Matlab	C	19 (PET)	MW, SC	20 (30.30%)
Jin et al. [22]	Yes	279 (CT) 279 (PET)	HSLM, GRM, GLRLM, GLCM, ARM, Wavelet	Mazda	OS	223 (CT) 234 (PET)	LASSO, 10-FCV	24 (36.36%)
Mesguich et al. [23]	Yes	87 (CT) 87 (PET)	SUVmax, FO, GLCM, GLRLM, GLDM, NGTDM, GLSZM	Pyradiomics	OS	2 (CT) 3 (PET)	RFT and correlation matrix	26 (39.39%)
Ripani et al. [24]	Yes	n.d. (PET)	SH, FO, GLCM, GLRLM, GLZLM, NGTDM	LifeX	OS	n.d. (PET)	MW	15 (22.73%)
Jamet et al. [25]	Yes	15 (PET)	GLCM, GLRLM, GLSZM	Pyradiomics	OS	5 (PET)	SC	39 (59.09%)
Xu et al. [26]	Not	N.A.	N.A.	N.A.	N.A.	N.A.	N.A.	14 (22.95%) **

* For original and oversampled database, respectively. ** Deep learning analysis. ARM: autoregressive model; C: commercial; FTs: features; GLCM: gray-level co-occurrence matrix; GLDM: gray-level difference matrix; GLRLM: gray-level run-length matrix; GLSZM: gray-level size zone matrix; GLZLM: gray-level zone-length matrix; GRM: gray-level absolute gradient; GLSM: gray-level scale matrix; HSLM: gray-level histogram; LASSO: least absolute shrinkage and selection operator; n.d.: not defined; N.A.: not applicable; NGLDM: neighborhood gray-level different matrix; NGTDM: neighborhood gray-tone difference matrix; OS: open source; RFT: random forest tree; ROC: receiving operating characteristics; RQS: radiomics quality score; SH: shape; Sw: software; TA: texture analysis.

**Table 3:** Summary of studies’ data mining.

Author	AI Area	AI Sw	Sw Class	Data-Mining Algorithms	Validation	Validation Test
Zhong et al. [17]	ML	R-software	OS	Cox, GB-Cox, CoxBoost, GBM, RSF, SVCR	Yes	5-FCV
Milara et al. [18]	ML	Orange	C	DT, SVM, RSF, LR, kNN, NN	Yes	5-FCV
Ni et al. [19]	ML	R-software	OS	n.d.	Yes	n.d.
Mannam et al. [20]	ML	Weka Data Mining, XLSTAT	C	Naive Bayesian, OneRules, single/multinomial LR, MLP, LM, RF, AdaBoost, Bagging, ICO, RaF, kNN, SVM, LogitBoost	Yes	10-FCV
Milara et al. [21]	ML	Orange	C	decision tree, SVM with linear polynomial and RBF kernels, RF, LR, kNN, and NN	Yes	5-FCV
Jin et al. [22]	ML	R-software, Python, IBM SPSS, and MedCalc	OS/C	Multivariate LR	Yes	10-FCV
Mesguich et al. [23]	ML	Scikit-learn library	OS	LR, GNB, kNN, SVM, RF	Yes	5-FCV
Ripani et al. [24]	N.A.	N.A.	N.A.	N.A.	N.A.	N.A.
Jamet et al. [25]	ML	Python	OS	RSF	Yes	5-FCV
Xu et al. [26]	DL ML	Python	OS	CNNs, V-Net, W-Net RF, kNN, SVM	Yes	3-FCV

AI: artificial intelligence; C: commercial; CNNs: convolutional neural networks; Cox: Cox proportional hazards model; CoxBoost: Cox model by likelihood based boosting; DL: deep learning; DT: decision tree; FCV: fold cross-validation; GB-Cox: linear gradient boosting models based on Cox’s partial likelihood; GBM: generalized boosted regression modelling; GNB: Gaussian Naive Bayesian; kNN: k-nearest neighbors; ICO: iterative classifier optimizer; LR: logistic regression; ML: machine learning; MLP: multilayer perceptron; N.A.: not applicable; n.d.: not defined; NN: neural network; RaF: randomizable filtered classifier; RBF: radial basis function; RF: random forest; RSF: random survival forest; SVCR: support vector regression for censored data model; SVM: support vector machine; Sw: software.

## Data Availability

Not applicable.
